# Prefrontal oxygenation during verbal fluency in severe obesity

**DOI:** 10.1007/s00702-026-03155-7

**Published:** 2026-04-22

**Authors:** B. Warrings, A.-C. Koschker, F. G. Schuler, F. Seyfried, S. Störk, M. Fassnacht, M. J. Herrmann

**Affiliations:** 1 Department of Psychiatry, Psychosomatics and Psychotherapy, University Hospital Würzburg, Margarete-Höppel Platz 1. 15, 97080 Würzburg, Germany; 2 Department Internal Medicine I, Division of Endocrinology and Diabetology, University Hospital Würzburg, Würzburg, Germany; 3 Department Clinical Research and Epidemiology, Comprehensive Heart Failure Center, University Hospital Würzburg, Würzburg, Germany; 4 Department General, Visceral, Transplant, Vascular, and Pediatric Surgery, University Hospital Würzburg, Würzburg, Germany; 5 Department of Internal Medicine I, Division of Cardiology, University Hospital Würzburg, Würzburg, Germany

**Keywords:** Prefrontal brain activity, Executive functions, Obesity, NIRS

## Abstract

Obesity is a highly prevalent chronic disease with major medical, psychosocial, and economic consequences worldwide. Beyond its association with metabolic and cardiovascular comorbidities, obesity is increasingly linked to impairments in executive functioning and alterations in prefrontal control networks. Neuropsychological and neuroimaging studies consistently indicate deficits in inhibition, cognitive flexibility, and executive control in overweight and obese individuals, accompanied by context-dependent changes in prefrontal activation under cognitive load. These findings underscore the relevance of investigating task-related prefrontal functioning in obesity using established executive paradigms. In this study, 40 healthy normal-weight controls (HC) and 43 patients with severe obesity (OB) were included, and task-related prefrontal activation was assessed with functional near-infrared spectroscopy (fNIRS) during performance of the verbal fluency task (VFT). On the behavioral level, patients with severe obesity showed reduced performance in the Stroop task assessing interference control and lower phonemic (letter) verbal fluency, with only a non-significant trend toward lower semantic (category) fluency during the VFT. On the neural level, these behavioral deficits were accompanied by reduced activation in prefrontal and frontotemporal regions, with attenuated left-lateralized oxygenation responses observed in patients across VFT conditions, and a reduced frontotemporal response most evident in the letter condition. Overall, the results highlight a close link between executive dysfunction and altered prefrontal activation patterns in severe obesity under cognitively demanding conditions.

## Introduction

Obesity is one of the most common chronic diseases in twenty-first century society. According to the World Health Organization, around 16% of adults (890 million) worldwide are living with obesity in 2022, with the trend continuing to rise (NCD-Risk 2024). Obesity poses a major challenge to healthcare systems worldwide, with an estimated annual cost (USA in 2019) of 286 billion US dollars, and the estimated costs will rise in the coming years (Okunogbe et al. 2022).

Obesity is associated with an increased risk of numerous somatic diseases. These include metabolic syndrome with type II diabetes mellitus and cardiovascular diseases (including hypertension, heart failure, arteriosclerosis), and certain malignant diseases, including colon, endometrial, breast, oesophageal, and renal carcinomas. Due to their increased prevalence and significant potential for harmful effects on health, these diseases are recognized as comorbidities of obesity (Guh et al. 2009).

In addition to somatic consequences, obesity is often associated with a further reduction in quality of life due to mental disorders such as depression and anxiety (Baumeister and Härter 2007). Impairments in everyday and professional life can promote social withdrawal tendencies and thus contribute to the development or maintenance of mental illness. Studies in which weight loss was associated with an improvement in depressive and anxiety-related symptoms show a direct link between weight change and mental well-being (Blaine and Rodman 2007).

In addition to the high prevalence, understanding the functional and neurobiological mechanisms of obesity is also becoming increasingly important. Obese individuals, for example, show limitations in executive functions. Beutel et al. (2006) showed reduced executive attention and intentional control in obese adults compared to normal-weight control subjects. Comparable deficits have also been demonstrated in overweight and obese children, particularly with regard to the inhibition of automatic or impulsive reactions, which can have a negative effect on eating behaviour (Blanco-Gómez et al. 2015). Recent neuropsychological studies confirm these findings and consistently show that inhibitory control and cognitive flexibility in particular may be impaired in overweight and obese individuals, with these deficits being interpreted as functional markers of prefrontal control processes (Ruiz-Molina et al. 2024; Saruco and Pleger 2021).

Findings from imaging studies support these results. In PET studies, Volkow et al. (2009) demonstrated a negative correlation between BMI and glucose metabolism in prefrontal regions at rest, while task-evoked effects vary by paradigm and context. Recent systematic reviews and meta-analyses of fMRI-based studies confirm that prefrontal control networks are functionally altered in obesity, particularly in the context of food-related inhibition and cue reactivity. However, there is no consistent pattern of general prefrontal hypoactivation, but rather a context-dependent change in activation and connectivity, modulated by factors such as hunger status and task requirements (Saruco and Pleger 2021; Vartanian et al. 2025). Batterink et al. (2010) showed reduced neural activation in overweight female adolescents during a food-related go/no-go task, as well as a negative correlation between body mass index and inhibitory control. A recent meta-analysis further summarizes neural responses to visual food cues as a function of weight and hunger state (Vartanian et al. 2025).

The hypothesis of prefrontal dysfunction in obesity is supported by further PET studies. Le et al. (2007) found significantly reduced postprandial activation of the left dorsolateral prefrontal cortex (DLPFC) in obese men, a region attributed with inhibitory control functions in eating behaviour. These findings were later confirmed in overweight women, with clear differences between normal-weight, formerly obese, and overweight study participants (Le et al. 2007), implying that obesity should not be understood exclusively as a metabolic disease but also affects cognitive and neural functions. Current functional near-infrared spectroscopy (fNIRS) studies support this assumption by demonstrating altered recruitment of the dorsolateral prefrontal cortex and inferior frontal gyrus in overweight and obese individuals, particularly during food-related inhibition and reappraisal tasks, suggesting reduced efficiency or compensatory over-recruitment of prefrontal control mechanisms (Parker et al. 2023; Svaldi et al. 2026).

In a therapeutic context, fMRI studies provide evidence of the relevance of executive control for treatment success. Goldman et al. (2013) examined patients after bariatric surgery and showed that successful weight loss is associated with increased activation of the DLPFC during inhibitory demands in response to food-related stimuli. This suggests that the mobilization of prefrontal control networks may be a relevant neurobiological component of long-term therapeutic success. This assumption is supported by recent fMRI and EEG studies showing that successful weight reduction or interventions may be associated with improvements in executive performance and normalization of prefrontal activation and connectivity patterns, further underscoring the clinical relevance of prefrontal control mechanisms for maintaining therapeutic effects (Li et al. 2023; Liu et al. 2025).

Given the consistently reported impairments in executive functions in obese individuals and the changes in prefrontal control networks demonstrated in recent imaging studies, a targeted investigation of prefrontal activation during executive tasks appears particularly useful. Recent work shows that prefrontal dysfunction in obesity occurs in a context- and task-dependent manner and manifests itself not at rest, but primarily under cognitive load. The Verbal Fluency Test (VFT) is an established executive task that requires coordinated recruitment of dorsolateral and inferior prefrontal areas and is therefore suitable for the functional assessment of prefrontal control mechanisms. It should be noted that previous fNIRS studies in obesity have predominantly examined overweight or moderately obese individuals, typically within a BMI range of approximately 30–40 kg/m^2^, and have mainly employed food-related or inhibitory control paradigms rather than classical executive tasks such as verbal fluency (e.g., Rösch et al. 2020; Cheng and Yang 2023; Parker et al. 2023; Svaldi et al. 2026). Consequently, the extent to which findings from these studies generalize to individuals with more severe forms of obesity remains unclear. In particular, higher BMI levels are associated with increased metabolic, vascular, and affective burden, all of which may influence both neural activation patterns and neurovascular coupling. To our knowledge, studies combining fNIRS with a verbal fluency paradigm in samples with severe or morbid obesity, especially at BMI levels comparable to the present sample, remain scarce. The current study therefore aims to extend existing research by investigating executive-related prefrontal activation during a VFT in a clinically severely obese population and by addressing a subgroup that is underrepresented in the existing neuroimaging literature.

Therefore, in this study, we will measure prefrontal activation during the VFT in patients with morbid obesity and compare it with a healthy, normal-weight control group to identify potential differences in task-related recruitment of prefrontal networks.

## Methods

### Participants

In total, the sample comprised 83 participants (40 healthy controls (HC) and 43 severely obese patients (OB)). The study was conducted in accordance with the latest version of the Declaration of Helsinki (2008) and was approved by the local ethics committee. All participants provided written informed consent.

The obese patients were participants in a larger study (WAS; German: *Würzburger Adipositas Studie*), an interdisciplinary project conducted at the Comprehensive Heart Failure Centre Würzburg (Koschker et al. 2022, 2023). The randomized study was initiated in 2011 and aimed to investigate the effects of weight loss through either gastric bypass surgery or psychotherapy-enhanced lifestyle intervention.

Inclusion criteria for obese patients were a body mass index (BMI) greater than 35 kg/m^2^ and an age of 18 years or older. Exclusion criteria included pregnancy or breastfeeding, unstable angina pectoris, metabolic disorders as the primary cause of obesity, alcohol or drug dependence within the past five years and long-term corticosteroid use. In addition to the general exclusion criteria of the WAS study, the following exclusion criteria for the NIRS measurement also had to be met: neurological disorders, impaired sensory functions. All participants had to be physically capable of performing the planned examination procedures, including a 6-min walk test.

Healthy control participants had to meet the inclusion criteria of an age of at least 18 years, normal body weight, and the absence of psychiatric or neurological disorders based on self-report.

The obese patient group consisted of 36 women and 7 men. Participants were between 20 and 61 years of age, with a mean age of 39,4 ± 11,1 years. The mean BMI was 49,3 ± 6,9 kg/m^2^, classifying the patients as severely obese.

The healthy control group consisted of 33 women and 7 men (no differences in sex distribution compared to obesity group: *χ*^2^(1) = 0,02, *P* = ,882). Participants were between 21 and 63 years of age, with a mean age of 40,0 ± 11,4 years (no differences compared to obesity group: *t*(81) =  − 0,24, *P* = ,815). Healthy controls had a significant lower mean BMI of 21,8 ± 1,9 kg/m^2^ compared to obesity group: (49,3 ± 6,9 kg/m^2^, *t*(81) = 24,34, *P* < ,001). Patients showed more depressive symptoms (*M*_*Ob*_ = 15,1 ± 7,0) than heathy controls (*M*_*HC*_ = 4,1 ± 4,2; *t*(81) = 8,62, *P* < ,001) measured with Beck depression inventory (BDI, Beck (1978)). Formal education was assessed based on the highest school-leaving qualification obtained within the German educational system. Lower and intermediate secondary school-leaving certificates were classified as intermediate education, whereas higher secondary school-leaving certificates were classified as higher education. In the patient group, 26 participants were classified as having intermediate education and 14 as having higher education; in the control group, 33 participants were classified as having intermediate education and 10 as having higher education. The distribution of educational levels did not differ significantly between groups (*χ*^2^(1) = 1,39, *P* = ,238).

### Verbal fluency task (VFT)

The Verbal Fluency Task (VFT) was conducted as described in previous studies (Herrmann et al. 2017; Katzorke et al. 2018) and included a letter version, a category version, and a control task, each lasting 30 s. Each task was presented as one block of 60 s, consisting of 30 s of activation followed by 30 s of rest, and was repeated three times alternating with the other tasks. The order of the task was always the same, we started with the letter task, continued with the category task and the control task. This sequence was repeated three times. General effects, such as fatigue, were distributed equally across all conditions. The VFT is less characterised by practice effects, as the task is so simple that even seriously ill patients can easily understand it. Since the NIRS hood is securely attached to the head, motion artefacts play a lesser role than, for example, in fMRI measurements. Our test participants were instructed to refrain from making any major movements, but this was not technically recorded.

In the letter version, participants were instructed to produce as many German nouns as possible beginning with the letters “A,” “F,” and “S” during the three runs, respectively. In the category version, participants were required to name German nouns belonging to the categories “animals,” “fruits,” and “flowers,” respectively. In the control task, participants were asked to repeatedly recite the days of the week at a moderate and continuous pace.

The number of correct verbal responses was counted and served as a measure of behavioural performance. Participants were instructed to remain silent during the resting phases, keep their eyes closed, and avoid head movements throughout the VFT.

### Functional near-infrared spectroscopy (fNIRS)

fNIRS data were acquired using the ETG-4000 Optical Topography System (Hitachi Medical Systems) with a 52-channel configuration. The system consists of a flexible plastic cap with 33 optodes positioned over the fronto-temporal cortex. Seventeen emitters and sixteen detectors were arranged in three rows (3 × 11 probe set). With an inter-optode distance of 3 cm, light penetration reached approximately 1,5–2,5 cm into cortical tissue (Hoshi et al. 2005).

The system emitted near-infrared light at a sampling frequency of 10 Hz using two wavelengths (695 ± 20 nm and 830 ± 20 nm), allowing for the measurement of changes in oxyhaemoglobin [OxyHb] and deoxyhaemoglobin [DeoxyHb]haemoglobin, with an increase in OxyHb and a corresponding decrease in DeoxyHb reflects cortical activation (Boas et al. 2004).

**Fig. 1:**
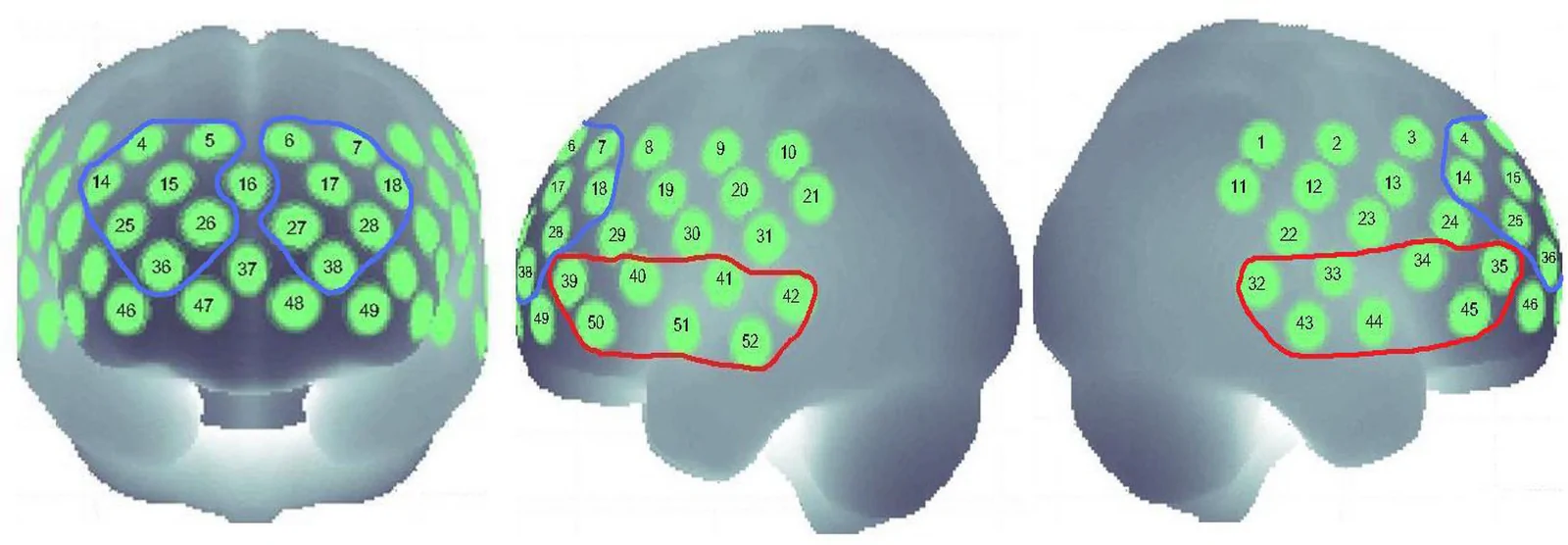
Position of the probe set over prefrontal brain areas, with the defined ROIs; the middle optode (not shown here) of the lowest row was positioned over Fpz

Probe placement was based on the international 10–20 EEG system (Jasper 1958). The central optode of the lower row (#26, located between channel 47 and 48 in Fig. 1) was positioned at electrode location Fpz, with the remaining optodes extending laterally toward positions T3 (left hemisphere) and T4 (right hemisphere).

### Neuropsychology

Additionally, executive interference control was assessed using the classical Stroop task with color-word cards (Bäumler 1985). Performance was recorded as the total time in seconds to name all items of the cards. Three different conditions (words, color, colored words) were repeated three times each. Interference was determined by subtracting performance of the incongruent color–word condition from the color-naming baseline condition, with more negative values indicating greater susceptibility to interference.

### Data analyses

#### Task performance

To assess VFT performance, the number of words produced in each task condition (letter, category, control) was counted and summed for each participant. Mean values of correctly produced words were calculated for each condition and group. Group differences were analysed using independent-samples *t*-tests, and 2-sided p values are reported.

#### Functional near-infrared spectroscopy

fNIRS data were pre-processed using a moving average with a 5-s time window to reduce high-frequency noise, a low-pass filter at 0,5 Hz, and a discrete cosine filter to remove signal drifts. Subsequently, time epochs were baseline-corrected using a 5-s pre-task interval and averaged across task repetitions for each participant and condition (Herrmann et al. 2008). For statistical analysis, baseline corrected mean haemoglobin concentration changes were calculated separately for each channel, condition, and participant over the 30-s activation period contrasted to the control condition. For statistical analyses, we calculated mean activity for both hemispheres with two regions of interest (ROI) each (see Fig. 1: frontotemporal cortex [FTC]: left side channel # 39, #40, #41, #42, #50, #51, #52; right side channel #32, 33, 34, 35, 43, 44, 45; frontomedial cortex [FMC] left side channel # 6, #7, #17, #18, #27, #28, #38; right side channel #4, #5, #14, #15, #25, #26, #36). This ROI definition and analyses, as well as laterality index, were based on a previous study (Katzorke et al. 2018). We analysed the data with an *ANCOVA* with the main factors hemisphere (left, right), ROI (FTC, FMC), group (obese patients [Ob], healthy controls [HC]), separately for both conditions. Due to group differences in depressive symptoms, we added BDI as covariate into the analyses. Both [OxyHb] and [DeoxyHb] are strongly interrelated and represent physiologically coupled aspects of the same underlying neurovascular response rather than independent biological processes. They therefore do not constitute independent outcome variables in a statistical sense, and analyzing them separately does not require correction for multiple comparisons across chromophores. At the same time, the two signals differ in their physiological sensitivity and functional specificity: [DeoxyHb] changes are typically more closely aligned with the BOLD signal and may provide higher spatial specificity, whereas [OxyHb] generally exhibits larger signal amplitudes and higher signal-to-noise ratio. Reporting both chromophores thus allows for a more comprehensive characterization of the hemodynamic response without inflating the number of independent statistical tests. To illustrate significant differences between healthy controls and patients in neural activation on a channel wise manner, we used *t*-tests between groups for each condition and channel for both chromophores ( [OxyHb], [DeoxyHb]).

## Results

### Behavioural results

Analysis of behavioural data during the VFT shows significantly lower performance in the “letters” condition for the patient group (*M*_*Ob*_ = 19,2 ± 6,0) compared to the healthy control group (*M*_*HC*_ = 24,3 ± 7,6; *t*[81] = 3,41, *P* < ,001) and also a tendency toward poorer performance in the “Categories” condition (*M*_*Ob*_ = 34,7 ± 8,2; *M*_*HC*_ = 37,6 ± 7,6; *t*[81] = 1,67, *P* < ,1). In the control condition, there were no significant differences between healthy subjects (*M*_*HC*_ = 57,1 ± 14,3) and the patient group (*M*_*Ob*_ = 60,1 ± 21,1; *t*[81] = -0,76, *P* = ,452). In the neuropsychological test (Stroop), the patients showed significantly greater interference and thus poorer performance (*M*_*Ob*_ = -33,3 ± 11,1) than the healthy control group (*M*_*HC*_ = -24,2 ± 11,4; *t*[81] = 3,71, *P* < ,001).

### Functional brain activation

For the analyses of the NIRS data*,* mixed repeated-measures ANCOVAs were conducted with side and ROI as within-subject factors, group as a between-subject factor, and BDI as a covariate, separately for [OxyHb] and [DeoxyHb], for both conditions (letter and category, contrasted with control condition).

*Letter condition of the VFT (see *Table 1*)*: For the main effect of ROI, significant changes were observed for both [OxyHb] (F(1, 80) = 20,06, p < ,001, ηp^2^ = ,200) and [DeoxyHb] (F(1, 80) = 11,25, p = ,001, ηp^2^ = ,123).

**Table 1:** fNIRS results of the letter condition in VFT

	[OxyHb]		[DeoxyHb]	
	*F* [1.80]	*P*	*F* [1.80]	*P*
Hemisphere	0.00	0.969	0.90	0.345
Hemisphere* BDI	0.99	0.324	0.08	0.773
Hemisphere * group	**5.33**	0.024*	0.08	0.783
ROI	**20.06**	< 0.001***	**11.25**	0.001***
ROI * BDI	0.34	0.564	0.02	0.883
ROI * group	**4.16**	0.045*	0.42	0.522
Hemisphere * ROI	0.24	0.624	0.01	0.907
Hemisphere * ROI * BDI	0.70	0.405	0.87	0.354
Hemisphere * ROI * group	1.26	0.265	1.63	0.205
BDI	0.20	0.654	0.12	0.731
Group	3.47	0.066	0.01	0.913

^*^P < 0,05, ***P < 0,001

A significant interaction between hemisphere and group was found for [OxyHb] ((1, 80) = 5,33, p = ,024, ηp^2^ = ,062). Likewise, the interaction between ROI and group was significant for [OxyHb] (F(1, 80) = 4,16, p = ,045, ηp^2^ = ,049).

All other within-subject interactions were non-significant. For the between-subject effects, neither BDI, nor group reached statistical significance.

*Category condition of the VFT (see *Table 2*)*: For the main effect of ROI, significant changes were observed for [OxyHb] (F(1, 80) = 5,93, p = ,017, ηp^2^ = ,069). A significant interaction between hemisphere and group was found for [OxyHb] (F(1, 80) = 6,07, p = ,016, ηp^2^ = ,071). Additionally, we found a three-way interaction between hemisphere × ROI × group reached statistical significance for [DeoxyHb] (F(1, 80) = 4,48, p = ,037, ηp^2^ = ,053), indicating that the combined effect of hemisphere and ROI differed between groups.

**Table 2:** fNIRS results of the category condition in VFT

	[OxyHb]		[DeoxyHb]	
	*F* [1.80]	*P*	*F* [1.80]	*P*
Hemisphere	0.21	0.651	2.14	0.147
Hemisphere* BDI	0.62	0.435	1.94	0.168
Hemisphere * group	**6.07**	0.016*	0.16	0.694
ROI	**5.93**	0.017*	0.80	0.375
ROI * BDI	0.73	0.397	1.45	0.232
ROI * group	2.63	0.109	0.02	0.890
Hemisphere * ROI	0.00	0.971	1.59	0.211
Hemisphere * ROI * BDI	0.01	0.919	3.74	0.057
Hemisphere * ROI * group	0.12	0.729	**4.48**	0.037*
BDI	0.06	0.808	2.39	0.126
Group	3.75	0.056	0.21	0.651

^*^p < 0,05, ***p < 0,001

No further interactions reached significance (*P* > ,05). For the between-subject effects, neither BDI, F(1, 80) = 2,39, p = ,126, ηp^2^ = ,029, nor group, F(1, 80) = 0,21, p = ,651, ηp^2^ = ,003, showed significant main effects.

To further analyse the hemisphere * group interaction effect for [OxyHb] in both conditions we calculated a laterality index between left minus right hemisphere for both regions and found lower left-sided activity in patients (letter: *M* = -0,01 ± 0,12; category *M* = -0,02 ± 0,09) compared to healthy controls (letter: *M* = 0,06 ± 0,18, *t*[81] = 2,21, *P* < 0,05, Cohen’s d = 0,49; category *M* = 0,03 ± 0,11,* t*[81] = 2,65, *P* < 0,01, Cohen’s d = 0,59). The laterality index correlates negatively with BMI for the total sample (r_pearson_ = -,25; p < ,05), but not for the performance during the VFT (category: r_pearson_ = 0; p = ,96; letter: r_pearson_ = ,16; p = ,14). The negative correlation indicates higher BMI with more negative laterality index (therefore less left sided activation). Further, the laterality index does not significantly correlate with BDI for the total sample (category: r_pearson_ = -,14, p = ,22; letter: r_pearson_ = -,09, p = ,42), suggesting that depressive symptom severity was not strongly associated with laterality at the bivariate level. Also, this does not rule out more subtle or non-linear effects, it does not support the assumption that the observed laterality pattern is primarily driven by any depressive symptoms.

To analyse the interaction effect ROI* group [OxyHb] in the letter version, we calculated the mean concentration of both hemispheres, separately for both ROIs. Healthy controls showed more activity in FTC (*M* = 0,49 ± 0,32) compared to patients (*M* = 0,30 ± 0,25,* t*[81] = 3,06, *P* < ,01), without significant differences in FMC (patients: *M* = 0,08 ± 0,16; healthy controls (*M* = 0,14 ± 0,16,* t*[81] = 1,71, *P* = 0,09).

**Fig. 2:**
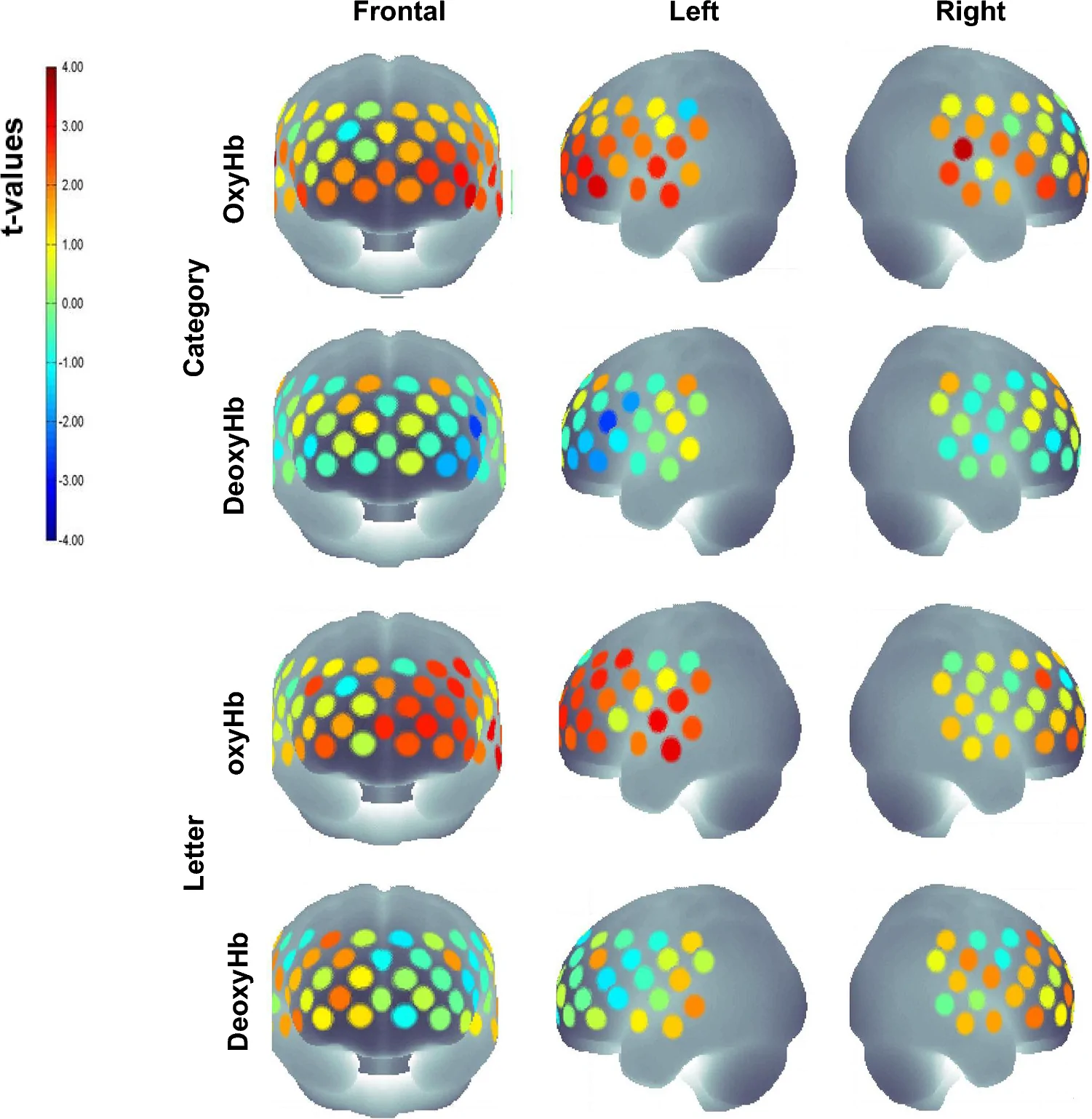
t-maps of the statistical comparison between healthy controls and patients for letter and category version of the VFT for [OxyHb] and [DeoxyHb]

For further illustration of the effects, we statistically compared (by using t-tests) healthy controls and patients for letter and category version of the VFT for [OxyHb] and [DeoxyHb]. Red colour in Fig. 2 shows higher concentrations in healthy controls, indicating higher activation for [OxyHb], while blue indicates higher activation in healthy controls for [DeoxyHb].

## Discussion

This study examined the neural and behavioural correlates of executive functions in patients with severe obesity and compared them with a healthy control group. At the behavioural level, the present sample with severe obesity (BMI > 35) showed both reduced performance in phonemic and semantic verbal fluency and increased interference in the Stroop test, indicating impairments in central executive control processes. These findings are consistent with an extensive literature describing executive function deficits in obesity, with inhibition and cognitive control in particular being reported as consistently affected (Smith et al. 2011; Yang et al. 2018). Meta-analytic evidence also shows that these deficits are more pronounced in obesity than in overweight individuals, underscoring the relevance of the observed effects in a clinically severely obese population (Yang et al. 2018).

The increased Stroop interference observed in our sample suggests a reduced ability to suppress automated responses under conditions of conflict, a mechanism that has repeatedly been discussed as impaired in theoretical models of obesity (Smith et al. 2011). Although Stroop paradigms have not been used consistently in obesity research, our results are consistent with findings that generally indicate impaired inhibitory control at higher BMIs (Yang et al. 2018).

The reduced performance in both variants in both phonemic and semantic verbal fluency tasks extends the existing literature on cognitive dysfunction in obesity.

Although executive deficits have been widely studied, few investigations, particularly in clinically severely obese samples, have distinguished between fluency domains. This differentiation is important, as phonemic fluency primarily relies on strategic search and executive control, whereas semantic fluency additionally depends on the organization and accessibility of semantic networks.

Impairments across both domains therefore suggest not only inhibitory deficits but broader disturbances in the integration of executive regulation and lexical–semantic retrieval. Given that verbal fluency reflects self-initiated, goal-directed cognitive organization, such deficits may have clinical relevance, as behavior-based obesity treatments require strategic planning, monitoring, and flexible adaptation.

The present findings showed significantly reduced performance in phonemic verbal fluency in individuals with obesity compared to normal-weight controls, while semantic fluency trend toward lower performance. This pattern suggests that the observed differences may primarily reflect difficulties in controlled lexical retrieval and strategic search processes rather than a generalized executive impairment. Phonemic fluency tasks require strategic search processes within the mental lexicon and the generation of retrieval strategies based on phonological cues (Shao et al. 2014). Neuroimaging evidence further supports this interpretation: functional near-infrared spectroscopy studies have demonstrated that phonemic verbal fluency strongly engages the dorsolateral prefrontal cortex and frontotemporal networks involved in controlled retrieval and cognitive control (Herrmann et al. 2017). In contrast, semantic fluency relies more strongly on the organization and accessibility of semantic memory and typically involves more automatic retrieval processes (Henry and Crawford 2004). The presence of a trend-level reduction in semantic fluency may indicate a subtle inefficiency in lexical access, whereas the stronger deficit in phonemic fluency points to greater involvement of controlled retrieval mechanisms. Given that obesity has been associated with alterations in prefrontal cognitive processes and executive control, potentially related to metabolic and inflammatory mechanisms affecting frontal brain networks (Yang et al. 2018), the present pattern may reflect obesity-related differences in controlled lexical search processes rather than a broad impairment of semantic knowledge.

In addition, unlike food-related paradigms, the present task does not depend on explicit appetitive cues or food-specific stimulus processing. This makes it possible to examine whether altered prefrontal recruitment in obesity is detectable beyond food-salient contexts and beyond relatively narrow forms of response inhibition.

In this context, the present findings contribute to a more differentiated understanding of prefrontal dysfunction in obesity. Rather than pointing only to a global reduction in prefrontal functioning, the observed effects suggest that obesity may be associated with differences in how prefrontal resources are distributed across hemispheres and regions during cognitive demanding, self-generated behavior. The results therefore extend prior work by indicating that altered prefrontal functioning in obesity may not be limited to cue-reactive or inhibitory processes, but may also affect the broader organization of executive control during verbal generation. At the same time, these findings should be interpreted cautiously as the present study cannot by itself establish a definitive mechanism. Rather, it provides evidence that verbal fluency may serve as a useful complementary paradigm for investigating the neural organization of prefrontal control in obesity.

To additionally control for potential effects of depressive symptoms, BDI was included as a covariate in the ANCOVA models. As no significant main effect of group was observed, the findings are better interpreted as not reflecting a generalized overall difference between groups. However, several significant interaction effects involving group were present, In the letter condition, significant group x hemisphere and group x ROI interactions were found for [OxyHb]. In the category task, significant group x hemisphere interactions for [OxyHb] and group x hemisphere x ROI interactions for [DeoxyHb] were observed. This pattern indicates that group-related differences are more appropriately interpreted as differences in the distribution of activation across hemispheres and regions of interest rather than as a simple main effect of group. Furthermore, the non-significant correlation between BDI and the laterality index does not suggest a strong linear relationship between depressive symptoms and laterality in the present sample. These findings support a differentiated interpretation of group related effects while taking depressive symptom burden into account.

Clinically, these findings are significant in that executive functions are central prerequisites for the implementation and maintenance of behaviour-based therapy requirements, such as planning, self-monitoring and flexible adaptation of behaviour. Existing studies show that better cognitive and executive performance is associated with higher therapy adherence and more favourable treatment outcomes, especially in the context of structured weight reduction or bariatric programmes (Spitznagel et al. 2013). At the same time, longitudinal studies suggest that cognitive functions may improve in part after substantial weight loss, which suggests a potential state dependency of the observed deficits and should be taken into account when interpreting the available cross-sectional findings (Gunstad et al. 2011).

Our findings of reduced prefrontal activation in obese individuals during a verbal fluency test (VFT) are consistent with the growing neurofunctional evidence pointing to reduced recruitment of prefrontal control networks in obesity. Rösch et al. (2020) previously demonstrated prefrontal hypoactivation in individuals with obesity and obesity with comorbid binge eating disorder across various experimental conditions, including an inhibitory control task, using fNIRS. The authors interpreted these reduced prefrontal responses as evidence of a general weakening of frontal control mechanisms, independent of specific eating disorder diagnoses (Rösch et al. 2020). Our results extend these findings by showing that comparable prefrontal hypoactivity also occurs during a verbal-executive retrieval process that places high demands on self-initiated generation, cognitive control, and monitoring.

More recent fNIRS studies also suggest reduced or altered prefrontal recruitment in overweight and obese individuals. Svaldi et al. (2026) report that the activation of prefrontal regions in response to food stimuli differs systematically between individuals who are overweight/obese, individuals with binge eating disorder, and individuals of normal weight, suggesting an altered functional integration of prefrontal control areas. In addition, Cheng and Yang (2023) show that food-related stimuli in particular are associated with reduced activation of prefrontal regions associated with executive functions. Although these studies do not use verbal executive tasks, they support the overarching model of functional vulnerability of the prefrontal cortex in obesity, which can manifest itself across different cognitive contexts.

One possible limitation of this study is the elevated severity of depressive symptoms in the patient group. Early studies showed that reduced prefrontal activation during the VFT can also be observed in depressed patients (Hermann et al., 2004). Similarly, depressed patients show poorer Stroop test performance (Verma et al., 2024). The prefrontal deficits described in this study could therefore be attributable to depressive symptoms.

Nevertheless, the reduced prefrontal activation observed during the VFT in our study may reflect a limited recruitment of executive neural resources. However, this interpretation should be made with caution. In addition to functional aspects of executive processing, vascular and metabolic factors may also contribute to reduced prefrontal activation. Obesity is associated with increased vascular burden and endothelial dysfunction, which may influence task-related hemodynamic responses during verbal fluency tasks measured with fNIRS (Heinzel et al. 2015). This assumption is supported by findings that prefrontal-mediated executive performance is not only related to neurofunctional activation patterns but also clinically relevant: Xu et al. (2017) showed that better prefrontal-mediated executive control, as measured by Stroop performance, is associated with more successful weight loss in overweight and obese adolescents and young adults. Taken together, these findings suggest that reduced prefrontal activation – as observed in our study during a VFT – is not only a neurocognitive marker of executive dysfunction but may also be associated with reduced behavioural regulation in a therapeutic context.

The present ANCOVA analysis involved testing a relatively large number of main and interaction effects, which is associated with an increased risk of Type I error. This constitutes a methodological limitation. However, the study was deliberately not restricted to a small number of isolated, strictly directional hypotheses, as one of its central aims was to explore and identify more complex and potentially unexpected patterns of association. Although a substantial reduction of the analyses to only a few pre-specified tests would have decreased the risk of false-positive findings, it would also have limited the opportunity to detect more differentiated effects and interactions. The findings should therefore be interpreted as exploratory and hypothesis-generating and should be examined more systematically in future studies. For this reason, p-values were not interpreted in isolation but were considered in conjunction with effect sizes and the substantive plausibility of the findings.

A further methodological limitation concerns the lack of counterbalancing of task order. Although the sequence of the letter task, the category task, and the control task was fixed, this sequence was repeated three times across the procedure. Accordingly, potential fatigue effects are less likely to have disproportionately affected one specific task, since the tasks were not represented only once in a single fixed position. However, because the order within each sequence remains constant, order effects such as practice, habituation, or carryover cannot be excluded. As a result, some of the observed differences may reflect not only the experimental manipulation itself but also the repeated sequential structure of the procedure. Future studies should therefore counterbalance or randomize task order to address this limitation more directly.

An important aspect when interpreting the present findings is the markedly elevated BMI of the patient sample. With a mean BMI of approximately 49 kg/m^2^, the current study extends previous fNIRS research, which has predominantly focused on overweight or moderately obese individuals (BMI ~ 30–40 kg/m^2^) and has mainly employed food-related or inhibitory control paradigms (e.g., Rösch et al. 2020; Cheng and Yang 2023; Parker et al. 2023; Svaldi et al. 2026). By contrast, the present study examines executive-related prefrontal activation during a verbal fluency task in a clinically severely obese population, thereby addressing a subgroup that has been largely underrepresented in the existing literature. Within this context, the observed pattern of reduced left-lateralized activation and diminished frontotemporal recruitment during the VFT can be interpreted as converging evidence for altered prefrontal engagement under cognitively demanding conditions. Notably, these neural differences are paralleled by impairments in phonemic fluency and Stroop performance, supporting the functional relevance of the observed activation patterns. While the absence of robust main group effects and brain–behaviour correlations suggests that these alterations are not uniformly expressed across all measures, the consistent interaction effects and laterality findings point to systematic differences in the organization of prefrontal processing rather than unspecific or purely vascular effects. At the same time, it cannot be excluded that metabolic, vascular, or affective factors associated with severe obesity modulate the hemodynamic response measured with fNIRS (Heinzel et al. 2015). However, such influences are inherent to the condition under investigation and may themselves be part of the pathophysiological mechanisms linking obesity and executive dysfunction. Taken together, the present findings provide evidence for altered, context-dependent recruitment of prefrontal control networks in severe obesity and extend prior work by demonstrating that these alterations are also observable during a verbal-executive paradigm.

In conclusion, the present findings suggest that severe obesity is associated with alterations in the context-dependent recruitment and lateralized organization of prefrontal control networks during executive processing, thereby extending existing neurofunctional models of obesity to a population with markedly elevated BMI. These alterations may have clinical relevance, as executive control processes are central to the implementation and maintenance of behaviour-based interventions, and could therefore represent a potential target for treatment optimization in severe obesity.

## Data Availability

The data that support the findings of this study are not openly available due to reasons of sensitivity and are available from the corresponding author upon reasonable request.
